# Ultrafast-UV laser integrating cavity device for inactivation of SARS-CoV-2 and other viruses

**DOI:** 10.1038/s41598-022-13670-8

**Published:** 2022-07-13

**Authors:** Sharad Ambardar, Mark C. Howell, Karthick Mayilsamy, Andrew McGill, Ryan Green, Subhra Mohapatra, Dmitri V. Voronine, Shyam S. Mohapatra

**Affiliations:** 1 Department of Medical Engineering, University of South Florida, USF Cherry Drive ISA 6049, Tampa, FL 33620 USA; 2 Department of Veterans Affairs, James A. Haley Veterans Hospital, Tampa, FL 33612 USA; 3 Department of Internal Medicine, Morsani College of Medicine, University of South Florida, 12901 Bruce B Downs Blvd. MDC 2511, Tampa, FL 33612 USA; 4 Department of Molecular Medicine, Morsani College of Medicine, University of South Florida, 12901 Bruce B Downs Blvd. MDC 2525, Tampa, FL 33612 USA; 5 Department of Physics, University of South Florida, Tampa, FL 33612 USA

**Keywords:** Biotechnology, Optics and photonics

## Abstract

Ultraviolet (UV) irradiation-based methods used for viral inactivation have provided an important avenue targeting severe acute respiratory-syndrome coronavirus-2 (SARS-CoV-2) virus. A major problem with state-of-the-art UV inactivation technology is that it is based on UV lamps, which have limited efficiency, require high power, large doses, and long irradiation times. These drawbacks limit the use of UV lamps in air filtering systems and other applications. To address these limitations, herein we report on the fabrication of a device comprising a pulsed nanosecond 266 nm UV laser coupled to an integrating cavity (LIC) composed of a UV reflective material, polytetrafluoroethylene. Previous UV lamp inactivation cavities were based on polished walls with specular reflections, but the diffuse reflective UV ICs were not thoroughly explored for virus inactivation. Our results show that LIC device can inactivate several respiratory viruses including SARS-CoV-2, at ~ 1 ms effective irradiation time, with > 2 orders of magnitude higher efficiency compared to UV lamps. The demonstrated 3 orders of magnitude cavity enhancement relative to direct exposure is crucial for the development of efficient real-time UV air and water purification systems. To the best of our knowledge this is the first demonstration of LIC application for broad viral inactivation with high efficiency.

## Introduction

To combat the outbreak of the severe acute respiratory-syndrome coronavirus-2 (SARS-CoV-2) global pandemic, various nanomaterials with tunable physical and chemical properties have been developed for vaccination and treatment of patients^1,2^. Also, photoelectrochemical oxidation assisted air-purifiers were developed as potential tools to control indoor SARS-CoV-2 exposure^3^. However, the pandemic viruses propagate through air and surfaces and hence there is a dire need to develop rapid and efficient methods to inactivate viral particles on surfaces and in the air, especially in public places such as hospitals, airports, stores, etc. against this pandemic and possible future pandemics.

Diverse light-mediated disinfection protocols are currently validated for surface, air, and water samples, as well as personal protective equipment^4^. UV irradiation as an effective, non-contact method of viral pathogen inactivation has been used for a long time, mainly in the form of low-pressure mercury lamps or light emitting diodes^5–8^. UVC light (in the range of 100–280 nm) has the largest antimicrobial and antiviral inactivation efficiency among the various UV ranges, including UVA (315–400 nm) and UVB (280–315 nm)^9^. The maximum absorption of nucleic acids is at ~ 265 nm, with UVC light causing damage by inducing photochemical fusion of two adjacent pyrimidines into covalently linked dimers, RNA–protein cross-linking, and site-specific molecular damage^10^. UV radiation has been explored for the treatment of human enteroviruses, Zika, hepatitis E, dengue, West Nile and others^11–19^, and, recently, for SARS-CoV-2^20–28^. The virucidal efficacy of UV light is influenced by a number of factors, including the target pathogen, environment, and material being decontaminated^29^. Further, germicidal UV has been combined with heat treatment for viral disinfection^30,31^, including SARS-CoV-2^32^. The problems with UV lamps include limited efficiency, requiring high power and long irradiation times (large doses), which is time-consuming and not suitable for use in many air conditioning systems, where the air passes by a UV lamp only for very short periods of time. The drawbacks of heating include low efficiency and large heat losses to environment, which require additional cooling efforts, and, hence, increase the cost of inactivation.

In addition, ultrashort laser pulses in the visible (Vis) at 425 nm and near-infrared (NIR) at ~ 800 nm ranges were reported to inactivate viruses^33–35^. It was suggested that impulsive stimulated Raman scattering, resulting in aggregation of viral capsid proteins, was the main inactivation mechanism^33^. However, the inactivation efficiency of the pulsed Vis–NIR irradiation is smaller than that of the germicidal UVC lamps. Pulsed UVB lasers, such as nanosecond excimer 308 nm laser, were also used for viral inactivation but showed low efficiency, similar to Vis–NIR^36^. High efficiencies were obtained using pulsed UVC lasers such as 193 nm excimer and 266 nm fourth harmonic Nd:YAG^37,38^. Nanosecond 266 nm UV pulsed laser irradiation revealed the nonlinear two-quantum mechanism of the RNA–protein crosslinking in inactivation of Venezuelan equine encephalomyelitis (VEE) virus with more than one order of magnitude increase of the quantum yield compared to the 254 nm UVC lamp^38^. This is contrasted with the linear one-photon nature of the conventional pyrimidine dimer formation mechanism which is present in both pulsed UV laser and UV lamp irradiation. Pulsed UV laser ablation is based on a combination of several mechanisms, including thermal and photochemical decomposition, that may increase viral inactivation efficiency beyond the conventional UV lamps. UV pulses contain more energy per unit time and can penetrate solutions further than continuous UV light^37^. On the other hand, UV pulses correspond to stronger absorption than Vis or NIR pulses, resulting in more effective inactivation.

Light absorption can be enhanced by coupling UV pulsed lasers to integrating cavities (ICs). Typical ICs have spherical geometry and walls made of highly reflective diffuse scattering materials^39,40^. The Lambertian light scattering from the walls generates uniform fields inside ICs and large effective optical path lengths, which have been used for sensing and spectroscopic applications^41–44^. Even though various UV boxes and cavities have previously been used for pathogen inactivation^45^, the virucidal properties of ICs have not been much explored. The diffuse scattering nature of ICs has an advantage over the specular scattering of the conventional cavities by providing a larger range of illumination angles that may reduce the shielding of viruses by microparticles. Therefore, we hypothesized that pulsed UV laser coupled to ICs can destroy virus more efficiently in reduced time. To test this hypothesis, we developed herein a new virus inactivation device based on a pulsed nanosecond 266 nm UV laser coupled to an integrating cavity, referred to as Laser Integrating Cavity Device (LICD). We examined inactivation of human coronaviruses, such as SARS-CoV-2^46^, and 229E Coronavirus (HCoV-229E)^47^, as well as respiratory syncytial virus (RSV)^48^. Our results show that LICD can inactivate 99.9% of the virus at ~ 1 ms effective total irradiation, which represents > 3 orders of magnitude increase in efficiency. To the best of our knowledge this is the first demonstration of using a pulsed UVC laser integrating cavity for the inactivation of human coronavirus, including SARS-CoV-2.

## Results

**Figure 1:**
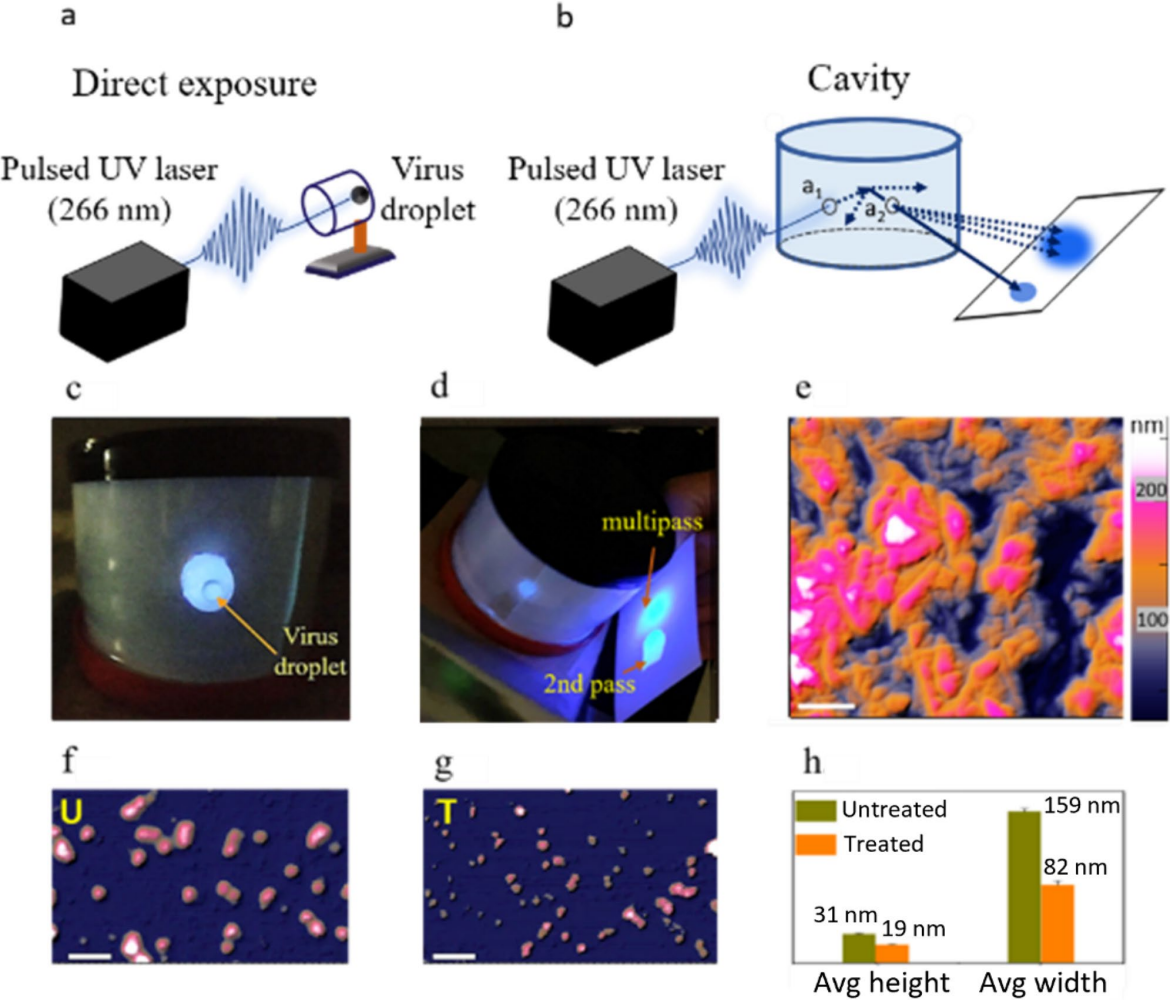
Virus inactivation using ultrafast UVC laser integrating cavity. (**a**) Schematic of the direct exposure of pulsed UVC laser on a droplet of virus solution in a vial. (**b**) Schematic of the LICD exposure of UVC laser irradiation on a droplet of virus placed inside the cavity at the location of aperture a_2_. (**c**) Photograph of the cavity with a virus droplet inside a vial. (**d**) Photograph of the cavity filled with UVC laser light and the reflection of fluorescence from the 2nd pass and multipass scattering as two bright spots on a white card, placed at the a_2_ aperture. (**e**) AFM height image of PTFE sheet used as a diffuse reflective LICD cavity wall. (**f**, **g**) AFM height images of the untreated (U) and UVC laser treated (T) HCoV-229E virions. Scale bar is 0.3 µm. (**h**) Average height and width of the untreated and treated HCoV-229E virions.

**Figure 2:**
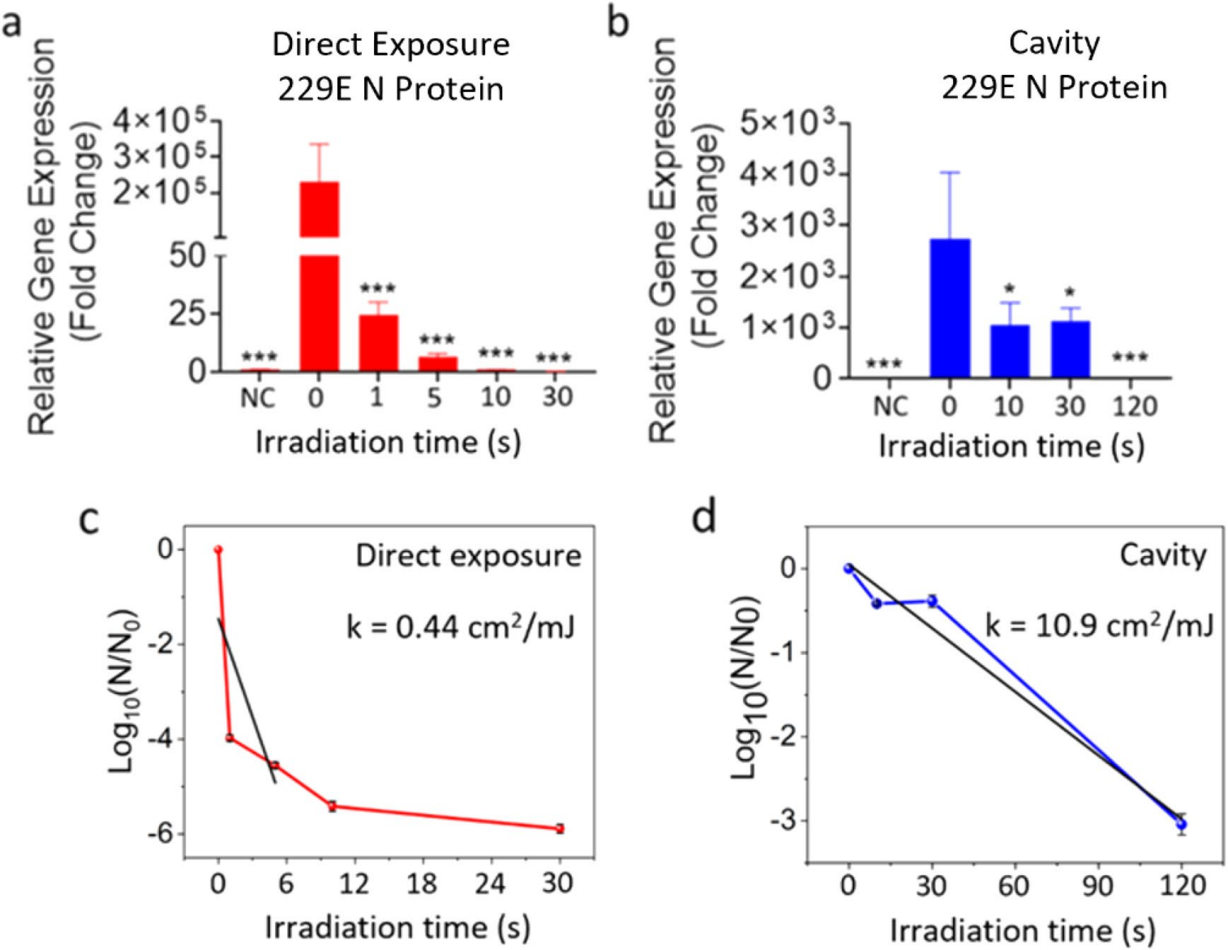
HCoV-229E virus was exposed to direct pulsed UVC laser (**a**) and cavity (**b**) for the indicated times. Calu-3 cells were treated 24 h after seeding with the indicated groups of 229E (3 MOI). At 72 h post-infection RNA was extracted and qPCR performed. Average fold change ± SEM, compared to the negative control (NC), is shown (N = 3). A 1-Way ANOVA and Dunnett’s post hoc test was used to determine significance compared to 0 s. HCoV-229E survival as a function of UV irradiation time via direct exposure (**c**) and cavity (**d**). *p < 0.05, **p < 0.01, ***P < 0.001, ****P < 0.0001.

Two different methods of laser exposure were investigated: (1) a direct exposure of the virus to the UV laser beam (Fig. 1a); (2) an indirect exposure of the virus to the UV laser irradiation when placed at a random location inside the LICD enclosure (Fig. 1b). We designed the LICD by coupling the 266 nm nanosecond pulsed UVC laser to a cylindrical IC enclosure made of highly UV reflective polytetrafluoroethylene (PTFE) coating^49,50^. Detailed schematics and spatial dimensions of LICD are shown in Figure S1. For the direct exposure, a plastic vial was placed horizontally as shown in Fig. 1a and a virus droplet was held at the bottom of the vial. For the LICD exposure, one vial was randomly placed at the bottom and the second vial was randomly placed on the side wall of the enclosure as shown in Fig. 1c.

After the incident laser beam is reflected from the inner wall of the enclosure, the diffusely scattered light undergoes multiple reflections, uniformly filling the whole volume. The diffuse scattering efficiency can be estimated by observing the brightness of the two fluorescence spots on a white card placed at the exit aperture of the cavity (Fig. 1d). The spot from the 2nd pass is directly reflected from the cavity wall by the incident laser beam. The spot from the multipass scattering is of similar brightness, indicating high diffuse reflectivity of the cavity walls. The PTFE coating has omnidirectional diffuse reflectivity of > 93% due to the porous structure.

We investigated the morphological properties of the IC walls using atomic force microscopy (AFM). Figure 1e shows the AFM height image of the PTFE sheet used as the reflective material for the IC. The observed irregularities are due to the presence of nanoparticles and pores that are highlighted by white dashed lines in Figure S2a. AFM analysis in Figure S2b showed the average particle size of 0.44 µm and the average pore size of 1.86 µm. AFM profiles of a typical particle and a pore are shown in Figures S2c and S2d, respectively. To observe the morphological changes caused by the pulsed UVC laser exposure, we performed AFM measurements on the untreated (non-irradiated) and treated (irradiated by direct pulsed UVC laser) HCoV-229E virions (Fig. 1f, g). The treated virus was irradiated for 30 min. The 30 min treatment was chosen to ensure the complete inactivation (more than 99.99%, see below). For the statistical analysis, ten virions were selected from both treated and untreated samples. The average height of the untreated virions was ~ 31 nm, while the treated virions had an average height of ~ 19 nm (Fig. 1h). The lateral width profiles showed the average width of the untreated virions ~ 159 nm and of the treated virions ~ 82 nm (Fig. 1h).

The AFM height profiles of the typical examples of the untreated and treated virions are shown in Figures S3a and S3b. These results indicate shrinkage of the viral particles after exposure to ultrashort UVC laser pulses, confirming the contribution of the ablation mechanism to virus inactivation.

To investigate coronavirus inactivation, we used a direct exposure of the 266 nm nanosecond UVC laser to inactivate the HCoV-229E virus. A 6 μl virus droplet in PBS was placed in a vial as described above. The laser irradiation times of 1, 5, 10 and 30 s correspond to the doses of 3.5, 17.6, 35.3 and 105.9 mJ/cm^2^. Upon the direct exposure of UVC laser, HCoV-229E replication was completely inactivated (99.9% reduction) after 4 s exposure, which corresponds to 15.6 ± 0.3 mJ/cm^2^ dose, measured using qPCR for 229E spike (S) (Figure S4a) and nucleocapsid (N) (Fig. 2a) transcripts after 72 h of infection in Calu-3 cells. To investigate the effects of the indirect exposure to the UVC laser on viral replication inside the IC enclosure, we performed the LICD exposure of HCoV-229E virus droplet in PBS placed inside a vial at two random positions inside the IC (shown in Figure S1) with the irradiation times of 10, 30, 120 and 1800s, which correspond to 0.05, 0.15, 0.6, and 9 mJ/cm^2^ doses, respectively. After the UV dose of 0.63 ± 0.02 mJ/cm^2^, HCoV-229E viral replication was inactivated (99.9% reduction), when measured by qPCR for 229E S (Figure S4b) and N (Fig. 2b) proteins after 72 h of infection in Calu-3 cells (Figure S5).

Figure 2c shows the log-linear survival plot of the direct inactivation kinetics, where N_0_ and N are the initial and final concentrations of the infectious viral units determined from the qPCR analysis. Only the linear part of the graph was fitted in agreement with the common approach^51^ as described in the Methods section. The linear regression fit resulted in the inactivation rate constant k = 0.443 ± 0.006 mJ/cm^2^. Figure 2d shows the LICD survival plot and the linear regression fitting with the inactivation rate constant k = 10.9 ± 0.4 mJ/cm^2^. These results indicate that both direct and LICD exposure to pulsed UVC laser eradicate viral ability to replicate in host cells. However, the LICD is an order of magnitude more effective as it requires a lower dose to achieve similar inactivation.

**Figure 3:**
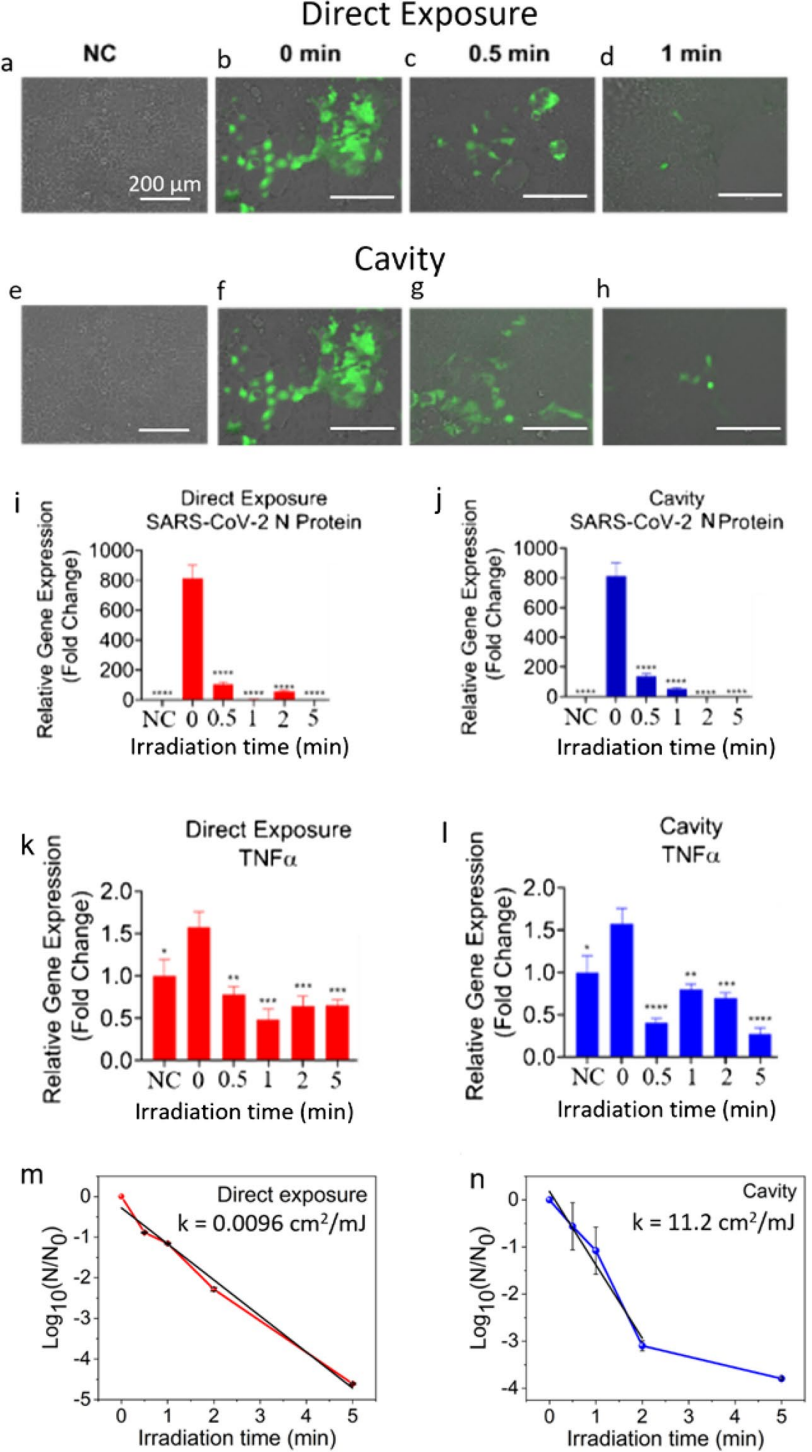
SARS-CoV-2 virus was exposed to indicated irradiation times of direct or cavity UVC laser light. Calu-3 cells were infected 24 h after seeding with the indicated groups of CoV-2 (0.1 MOI). (**a**–**h**) Images were taken 48 h post-infection using the EVOS microscope (Thermo Fisher). 200X. Scale bar = 200 µm. (**i**–**j**) SARS-CoV-2 N protein expression in Calu-3 cells. At 72 h post-infection RNA was extracted and qPCR performed. Average fold change ± SEM, compared to the negative control (NC), is shown (N = 3). A 1-Way ANOVA and Dunnett’s post hoc test was used to determine significance compared to 0 s. (**k**–**l**) Inflammatory marker expression in Calu-3 cells after SARS-CoV-2 infection. For the negative control, CoV-2 was exposed to UVC light under a handheld wand for 2 min. At 48 h post-infection RNA was extracted and qPCR performed. Average fold change ± SEM, compared to the negative control (NC), is shown (N = 3). A 1-Way ANOVA and Dunnett’s post hoc test was used to determine significance compared to 0 s. SARS-CoV-2 survival as a function of irradiation time via direct exposure (**m**) and cavity (**n**). *p < 0.05, **p < 0.01, ***P < 0.001, ****P < 0.0001.

Next we performed a direct exposure of the 266 nm nanosecond UVC laser to inactivate SARS-CoV-2. We used a 23 μl droplet of virus in PBS placed in a vial for the direct and LICD exposures as described above. The irradiation times of 30, 60, 120 and 300 s correspond to doses of 105.9, 211.8, 423.6 and 1059 mJ/cm^2^, respectively. Upon the direct exposure of UVC laser, SARS-CoV-2 replication was inactivated (99.9%) after 3 min and 715 mJ/cm^2^ dose exposure, measured using fluorescent microscopy (Fig. 3a–d) and qPCR for SARS-CoV-2 S (Figure S6a) and N (Fig. 3i) proteins after 48 h of infection in Calu-3 cells. We then performed the LICD exposure of SARS-CoV-2 with the irradiation times of 30, 60, 120 and 300 s corresponding to exposure doses of 0.15, 0.3, 0.6 and 1.5 mJ/cm^2^, respectively. After the UV dose of 0.60 ± 0.02 mJ/cm^2^, viral replication was inactivated (99.9% reduction), when measured using fluorescent microscopy (Fig. 3e–h) and qPCR for SARS-CoV-2 S (Figure S6b) and N (Fig. 3j) transcripts after 48 h of infection in Calu-3 cells. SARS-CoV-2 infection usually results in a pathological increase of the inflammatory protein TNF-α^46^. However, after exposure to both the direct (Fig. 3k) and LICD (Fig. 3l) UVC laser irradiation, SARS-CoV-2 infection causes significantly less TNF-α expression in Calu-3 cells. The linear regression analysis of the survival curve for the direct exposure in Fig. 3m resulted in the inactivation rate constant of k = 0.00965 ± 0.00004 mJ/cm^2^. The linear regression fit for the LICD in Fig. 3n resulted in the inactivation rate constant of k = 11.2 ± 0.1 mJ/cm^2^. This showed that LICD was three orders of magnitude more efficient than the direct exposure.

**Figure 4:**
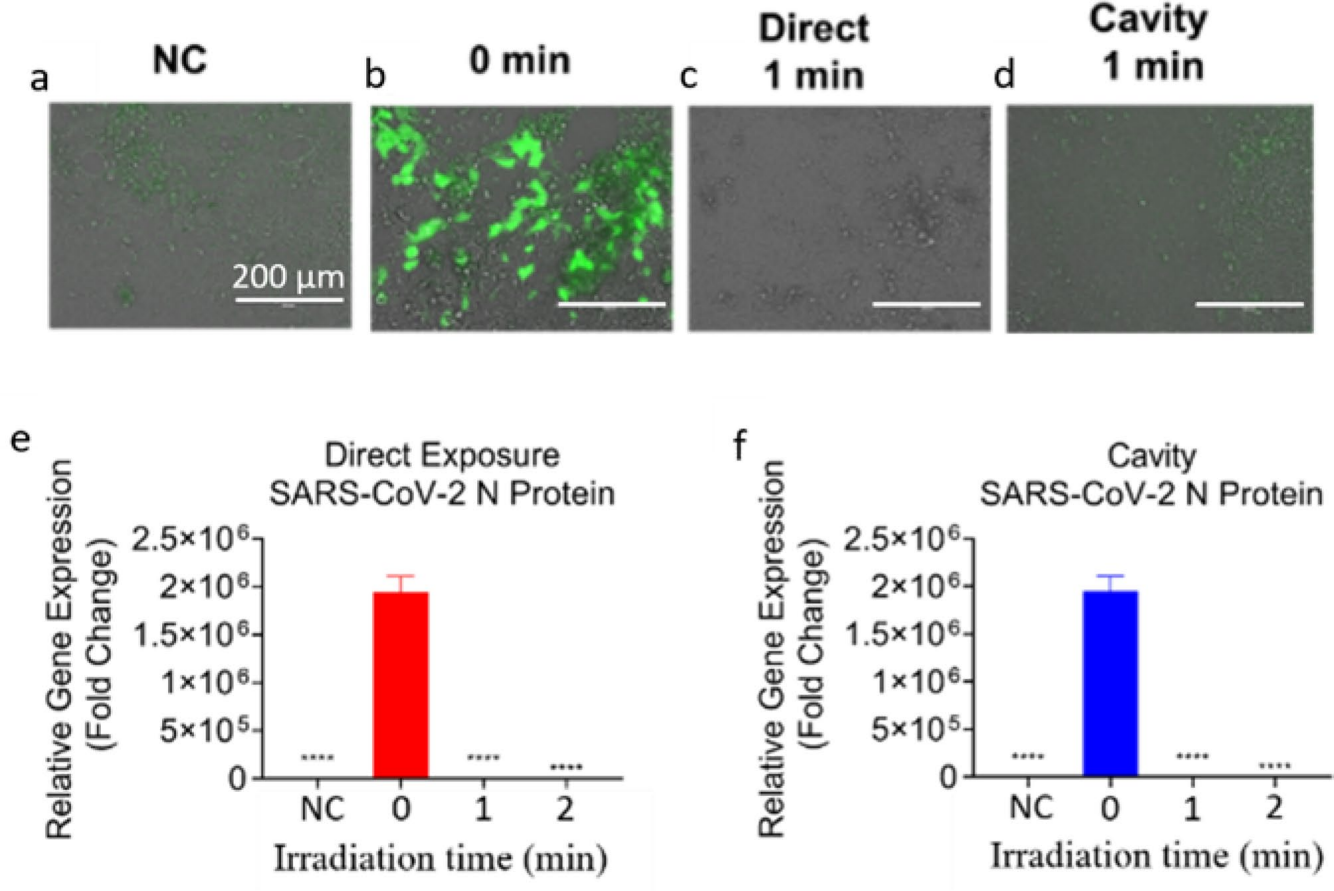
Reinfection of Calu-3 cells with culture supernatant from SARS-CoV-2 infected Calu-3 cells that were exposed to direct or cavity UVC laser light (Fig. 3). (**a**–**d**) Images were taken 48 h post-infection using EVOS microscope (Thermo Fisher). 200X. Scale bar = 200 µm. (**e**–**f**) SARS-CoV-2 N protein expression in Calu-3 cells. At 48 h post-infection RNA was extracted and qPCR performed. Average fold change ± SEM, compared to the negative control (NC), is shown (N = 3). A 1-Way ANOVA and Dunnett’s post hoc test was used to determine significance compared to 0 s. *p < 0.05, **p < 0.01, ***P < 0.001, ****P < 0.0001.

We next used the Calu-3 cell culture supernatant from the previous experiment to reinfect a new batch of Calu-3 cells with SARS-CoV-2. The cell culture supernatant containing the SARS-CoV-2 virus was exposed to the direct laser or LICD to inactivate the virus. The laser irradiation times were 60 and 120 s with 211.8 and 423.6 mJ/cm^2^ doses for direct exposure and 0.3 and 0.6 mJ/cm^2^ for LICD. Upon the direct exposure to the UVC laser, SARS-CoV-2 replication was completely inactivated at 211.8 mJ/cm^2^ dose, measured using fluorescent microscopy (Fig. 4a–c) and qPCR for SARS-CoV-2N (Fig. 4e) transcript after 48 h of infection in Calu-3 cells. After the UV dose of 0.6 mJ/cm^2^, viral replication was inactivated in LICD, when measured using fluorescent microscopy (Fig. 4d) and qPCR for SARS-CoV-2N (Fig. 4f) transcripts after 48 h of infection in Calu-3 cells. These results show that after exposure to the direct pulsed UVC laser or LICD, coronavirus samples, including SARS-CoV-2, are no longer able to cause a productive infection in cultured cells.

To test LICD on a different respiratory virus, we next exposed RSV virus expressing red fluorescent protein (RSV-RFP) to direct 266 nm nanosecond pulsed UV laser with irradiation times of 1, 5 and 15 s with corresponding doses of 3.5, 17.6 and 52.9 mJ/cm^2^ (Figures S7a-h). A dose of 17.6 mJ/cm^2^ resulted in a complete viral inactivation in Hep2 cells at 72 h post infection when observed by fluorescent microscopy. RSV-RFP virus was then exposed to the LICD, which successfully inactivated RSV-RFP in Hep-2 cells at 72 h of infection when exposed to doses as small as 0.3 mJ/cm^2^, however, a dose of 0.05 mJ/cm^2^ also inactivated most of the virus (Figures S7i-p).

As a control measurement, we also directly exposed the RSV-RFP virus to a 337 nm pulsed nanosecond UVB laser (Figure S8). The UVB laser irradiation had no inhibitory effect on the RSV-RFP viral replication in Hep-2 cells. These results confirm the use of UVC pulsed laser radiation for viral inactivation. We performed additional control measurements using two different UVC lamp sources for viral inactivation, Stratalinker unit (Figure S9) and a Handheld Wand (Figure S10). Both UVC lamps were able to inactivate the RSV-RFP viral replication in Hep2 cells at 72 h post-infection with irradiation time of 5 s and a dose of 68.5 mJ/cm^2^, when observed by fluorescent microscopy. However, two orders of magnitude higher inactivation efficiency was obtained using the LICD (dose of 0.6 mJ/cm^2^, Table 1).

**Table 1:** Linear regression parameters from fitting of the survival curves for the direct pulsed UVC laser and LICD cavity exposures of the HCoV-229E and SARS-CoV-2.

Virus	Exposure	*K* (mJ/cm^2^)	D_*90*_ (mJ/cm^2^)	D_99_ (mJ/cm^2^)	D_99.9_ (mJ/cm^2^)	Cavity enhancement
HCoV-229E	Direct	0.44 ± 0.01	5.1 ± 0.1	10.4 ± 0.2	15.6 ± 0.3	25
	Cavity	10.9 ± 0.4	0.21 ± 0.01	0.42 ± 0.02	0.63 ± 0.02	
SARS-CoV-2	Direct	0.00965 ± 0.00004	238 ± 1	476 ± 2	715 ± 3	1160
	Cavity	11.2 ± 0.1	0.201 ± 0.003	0.41 ± 0.01	0.60 ± 0.02	

Cavity enhancement factors show the increase of the inactivation efficiency of the LICD compared to the direct exposure.

Table 1 presents the results of the linear regression analysis of the HCoV-229E and SARS-CoV-2 inactivation kinetics. The inactivation rate constants k were obtained from the survival curves described in the Methods section. Table 1 also shows the UVC dose needed to inactivate 90% (D_90_), 99% (D_99_), and 99.9% (D_99.9_). D_99.9_ represents the “complete inactivation” requiring 715 mJ/cm^2^ for SARS-CoV-2 using the direct exposure, while only 0.6 mJ/cm^2^ using the LICD. This difference is quantified as the cavity enhancement factor (EF) of 1160 (Table 1) given by the EF equation in the Methods section.

The complete inactivation (99.9%) of HCoV-229E required 15.6 mJ/cm^2^ for the direct exposure, while only 0.63 mJ/cm^2^ was needed using the LICD (Table 1), resulting in the EF of 25 due to the cavity. The inactivation rate constant for the LICD of k = 10.9 mJ/cm^2^ was similar to that of SARS-CoV-2 for LICD. However, a larger rate constant of k = 0.44 mJ/cm^2^ for the direct pulsed UVC exposure of HCoV-229E was obtained compared to k = 0.01 mJ/cm^2^ for SARS-CoV-2. This difference could be attributed to a smaller droplet size (6 μl) and structural and morphological differences.

## Discussion

The obtained inactivation rate constant for LICD exposure k = 11.2 mJ/cm^2^ is more than an order of magnitude larger than the previously observed rate constants for most ssRNA viruses using UVC lamps at 254 nm and ~ 5 times larger than that predicted for SARS-CoV-2^51^. On the other hand, the observed k = 0.01 mJ/cm^2^ for the direct exposure is by an order of magnitude lower than the typical literature values for other ssRNA viruses using 254 nm lamps. This difference could be explained by the different experimental conditions such as the relatively large droplet size of the SARS-CoV-2 solution (23 μl) and absence of stirring. These effects could lead to light attenuation in the sample and lower k values. Note that these effects, however, do not affect the EF values, which show the increase of the inactivation efficiency due to the IC effect.

A major difference between the UVC lamp and a pulsed UVC laser exposure from the applications point of view is in the effective irradiation time, which is much shorter for the pulsed laser. The total duration of laser exposure per unit time may be obtained by multiplying the nanosecond pulse duration by the pulse repetition rate. This results in the effective total irradiation time to inactivate both HCoV-229E and SARS-CoV-2 coronaviruses of ~ 1 ms using LICD, while only ~ 0.1 ms to inactivate RSV. Similar inactivation times are required for the direct exposure but with larger doses due to the difference of the illumination areas of the direct (~ 0.2 cm^2^) compared to the LICD (~ 200 cm^2^). Due to the differences in the genome organization and composition of the non-structural proteins these three viruses allow us to demonstrate the broad range inactivation ability of the pulsed UVC laser and LICD.

## Conclusions

In these studies, we performed fast inactivation of viruses using a pulsed 266 nm nanosecond UVC laser toward developing a broad anti-viral LIC device. To test this novel system, we used 3 different types of viruses including HCoV-229E, RSV-RFP and SARS-CoV-2. A comparison of the LICD, fabricated using a highly UV diffuse scattering material PTFE, with the direct exposure of the UVC laser beam, demonstrated efficient viral inactivation after exposure to LICD. The time needed for inactivation might be further reduced by increasing the laser power, by using additional optical elements, and improving the diffuse reflecting properties of the device with more advanced reflective materials. Also, beyond viral inactivation, the application of LICD may be extended to other pathogens such as bacteria^52–54^ and mold^55^. The technological progress in the development of UV lasers is envisioned to reduce the cost and make the LICD technology widely available in the near future. The application of LICD to air and water purification systems will benefit from the increased inactivation efficiency. LICD-based air conditioners may be used in enclosed spaces such as airplanes, stores and offices. LICD-based water purification systems may be used in household and industrial settings. PTFE-coated water pipes would be useful in conjunction with their anti-fouling properties for wastewater treatment^56^.

The LICD may be used in public places due to the enclosed protection of the UVC exposed volume by the cavity. In general, exposure of skin to UVC radiation may cause oxidative damage by either the direct absorption of UV or by the reactive oxygen species^57^. However, it has been reported that low doses of far-UVC light (207–222 nm) may be harmless to the exposed human tissues^7^. Additionally, aluminum nanoparticles were proposed as shielding agents in a quantum medical approach based on UV radiation to significantly decrease the risk of photodamage of healthy tissues, while inactivating pathogens^58^.

## Materials and methods

### Cell culture

Cell lines (Calu3 and Hep2) were purchased from the American Type Culture Collection (ATCC, Virginia USA) and passaged no more than 25 times. Cells were cultured in a humidified incubator at 37 °C in a 5% CO_2_ (Carbon Dioxide) atmosphere. Cells were cultured in tissue culture-treated plates in the appropriate complete cell culture media [HEP2 = DMEM (GE Healthcare) containing 10% fetal bovine serum (FBS) (Atlanta Biologicals) and 1% penicillin/streptomycin (GE Healthcare)] [Calu3 = MEM (GE Healthcare) containing 20% FBS, 1% non-essential amino acids (GE Healthcare), 1% 100 mM sodium pyruvate (Gibco), and 1% penicillin/streptomycin].

### Viral infection

Three different viruses were used in these experiments. Red fluorescent protein (RFP) expressing respiratory syncytial virus (RSV-RFP), human coronavirus 229E (HCoV-229E), and SARS-CoV-2. HCoV-229E was obtained through BEI Resources, NIAID, NIH: Human Coronavirus, 229E, NR-52726. SARS-CoV-2 was provided to us by Dr. Pei-Yong Shi from the University of Texas Medical Branch, Galveston, TX, USA^59^. The strain of RSV used in all the experiments (RSV-RFP) was a recombinant A2 strain expressing a red fluorescent marker, mKate2, as well as the F protein from the clinical strain Line 19 (rA2-KL19F). Handling and storage of RSV-RFP and HCoV-229E, including all experimental procedures, were performed in a biosafety level 2 (BSL-2) laboratory. Handling, storage, and experiments using SARS-CoV-2 were performed in a BSL-3 laboratory. For all experiments, a monolayer of Hep2 (RSV-RFP) or Calu3 (SARS-CoV-2 and HCoV-229E) cells at 80% confluence was infected with various concentrations of the indicated virus MOI (as indicated in the figure legends). Cells were then incubated with the viral inoculum in Opti-MEM (Life Technologies) for 2 h at 37 °C. After this, the infectious medium was replaced by fresh growth media. Fluorescent microscopy (Keyence BZ-X800 or EVOS) was used to image cells and then the cell pellets were collected for RNA analysis.

### Quantitative reverse transcriptase PCR (qPCR)

Total cellular RNA was extracted from cell pellets using Trizol (Thermo Fisher Scientific) according to the manufactures' protocol. RNA was quantified using the Nanodrop (Thermo Fisher Scientific). One microgram of RNA was then reverse transcribed using the Maxima cDNA Reverse Transcription Kit (Thermo Fisher Scientific) according to the manufactures' protocol. qPCR performed on the cDNA was used to quantitate the relative expression levels of certain genes to β-actin as a control. Real time analysis was performed using BlazeTaq SYBR Green qPCR Mix 2.0 (Genecoepia), according to the manufactures' protocol, in a Bio Rad CFX-384 thermocycler using primers obtained from Integrated DNA Technologies (IDT). The data was analyzed using ΔΔCt calculations and expression of all genes was normalized to β -actin expression as a housekeeping gene. Average fold change ± SEM, compared to control, was then calculated. Data analysis was performed using the CFX Maestro software (Bio-Rad).

### Integrating cavity

We constructed the cylindrically shaped integrating cavity (IC) using a high reflectance porous PTFE sheet (Thorlabs) of 0.75 mm thickness which showed UVC reflectance of > 93%. The schematic diagrams and spatial dimensions are shown in Figure S1.

The optical path increase in the IC may be estimated using the approach of Fry, et al.^39,42^. The average distance between reflections inside an integrating cavity $\overline{d }$ = 4.1 cm is given by $\overline{d }$ = 4$\frac{V}{S}$, where *V* is the cavity volume, and *S* is the surface area. The average path length inside the IC, L = 63 cm was calculated by *L* = 4$\frac{V}{S(1-\rho )}$, where $\rho$ = 0.935 is the IC reflectivity estimated from the reflectivity of PTFE at 266 nm. The cavity enhancement factor (EF) for each virus was calculated by EF = $\frac{{k}_{cavity}}{{k}_{direct}}$, where ${k}_{direct}$ and ${k}_{cavity}$ is the inactivation rate constant for the direct and LICD exposure, described below.

### UV irradiation

The following UV sources were used: (i)* Pulsed UVC source* was 266 nm Nd:YAG nanosecond pulsed laser (JDS Uniphase NanoLaser™) with 1 mW average power, ~ 1 ns temporal pulse duration, and 10 kHz repetition rate. (ii) *Pulsed UVB source* was 337 nm nitrogen laser (VSL-337ND) with 5.2 mW average power, < 4 ns temporal pulse duration, and 10 Hz repetition rate. (iii) *Cw UVC lamp* (Stratalinker® UV Crosslinker 1800) had 5 bulbs, 8 W each, with 254 nm wavelength. (iv) *Cw UVC lamp* (Handheld Wand, Clear-Raze™) had a bulb of 18 W with 254 nm wavelength.

### Inactivation kinetics

The results of inactivation experiments were analyzed using the qPCR relative gene expression data (Figs. 2 and 3). The linear regression parameters in Table 1 were calculated using the first order kinetics given by ln(N/N_0_) = −*k* D, where N/N_0_ is the survival fraction, N is the relative gene expression at each UV dose (D), and N_0_ is the relative gene expression at zero dose. The experiments were carried out in 3 replicates for HCoV-229E and 4 replicates for SARS-CoV-2. The inactivation rate constant, *k* (mJ/cm^2^), was calculated for each virus strain and for both direct and LICD exposures. The load reduction doses to inactivate 90% (D_90_), 99% (D_99_) and 99.9% (D_99.9_) of the virus are given by, D_90_ = $\frac{-\mathrm{ln}[1-0.9]}{k}$, D_99_ = $\frac{-\mathrm{ln}[1-0.99]}{k}$, and D_99.9_ = $\frac{-\mathrm{ln}[1-0.999]}{k}$.

### AFM measurements

Viral drops were inactivated using 10% phosphate-buffered formalin for 20 min and washed using phosphate-buffered saline (PBS). Atomic force microscopy (AFM) measurements (OmegaScopeR, Horiba Scientific) were performed using a Si tip (Micromash) in the tapping mode with 20 nm average tip-sample distance on virus deposited on SiO_2_/Si substrate by drop casting. A 2 µm*2 µm scanning area was chosen for AFM, which comprised of several virions, out of which 10 virions were selected to calculate and compare the height and the width of the treated and untreated virions.

### Statistics

Experiments have been repeated three times with three replicates in each experiment. When comparing multiple groups, statistical significance for each experiment was determined using Analysis of variance (ANOVA) and the Dunnett’s post hoc test, *p < 0.05, **p < 0.01, ***P < 0.001, ****P < 0.0001. Calculations were performed and graphs produced using Prism 6.0 software (GraphPad). Graphs of results show the mean and error bars depict the mean plus or minus the standard error of the mean.

## Supplementary Information

Supplementary Information.

## Data Availability

The datasets generated and/or analyzed during the current study are available by request from a corresponding author.
