# Supplementary material for: Ultrafast-UV laser integrating cavity device for inactivation of SARS-CoV-2 and other viruses
Source: Sci Rep. 2022 Jul 13;12:11935. doi: 10.1038/s41598-022-13670-8 (PMC9279343; doi:10.1038/s41598-022-13670-8)
Supplement: Supplementary file 1 — Supplementary Information. [file 41598_2022_13670_MOESM1_ESM.docx]

Supplementary Information

**Ultrafast-UV Laser Integrating Cavity Device for Inactivation**

**of SARS-CoV-2 and Other Viruses**

Sharad Ambardar^1#^, Mark C Howell Jr^2,3#^, Karthick Mayilsamy^4#^, Andrew McGill^2,3,4^, Ryan Green^2,3^, Subhra Mohapatra^2,4*^, Dmitri V. Voronine^1, 5*^ and Shyam S Mohapatra^2,3*^

^1^*Department of Medical Engineering, University of South Florida, Tampa, FL 33620, USA*

^2^*Department of Veterans Affairs, James A. Haley Veterans Hospital, Tampa, FL 33612, USA*

^3^*Department of Internal Medicine, Morsani College of Medicine, University of South Florida, Tampa, FL 33612, USA*

^4^*Department of Molecular Medicine, Morsani College of Medicine, University of South Florida, Tampa, FL 33612, USA*

^5^*Department of Physics, University of South Florida, Tampa, FL 33612, USA*

^#These authors contributed equally to this work^

^*Corresponding Authors (Smohapa2@usf.edu; 813-974-4127; 12901 Bruce B Downs Blvd. MDC 2525 Tampa, FL 33612 USA); (Voronine@usf.edu; 813-974-7793; USF Cherry Drive ISA 6049 Tampa, FL 33620 USA; (Smohapat@usf.edu; 813-974-8568; 12901 Bruce B Downs Blvd. MDC 2511 Tampa, FL 33612 USA)^


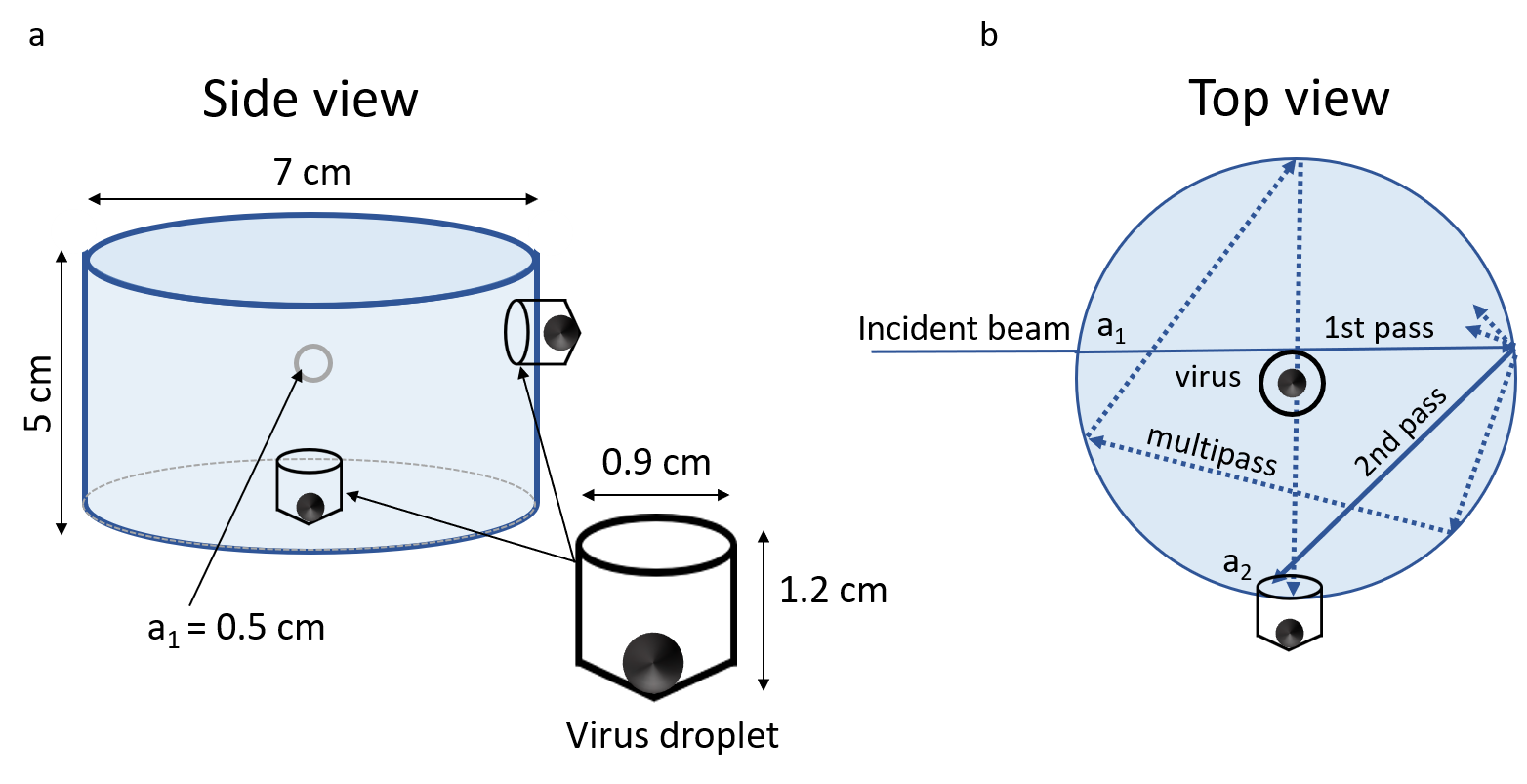


Figure S1. Schematic of the integrating cavity. (a) Side view shows cavity dimensions, laser beam entrance aperture a_1_ and two plastic vials containing virus droplets, one at the bottom of the cavity and one on the side. (b) Top view shows the schematic of laser beam scattering inside the cavity. A vial with a virus droplet placed at the aperture a_2_ is illuminated by multiply scattered beams: the 2^nd^ pass (solid line) and one of the multipass beams (dashed line) formed by diffuse scattering inside the integrating cavity are shown.


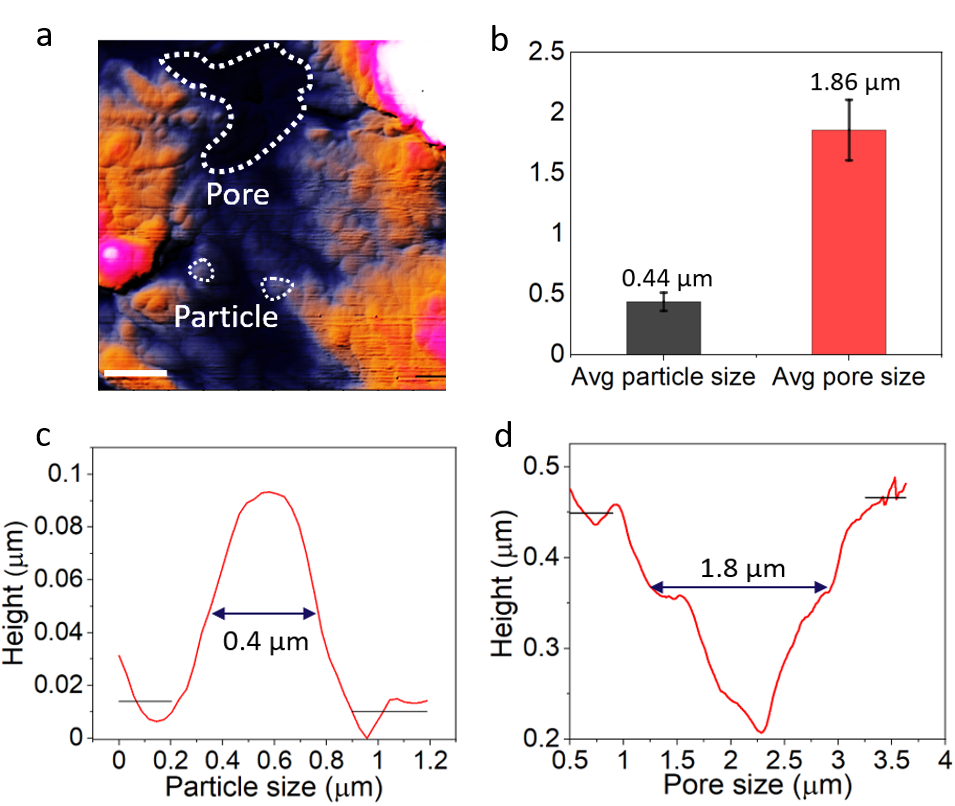


Figure S2. Atomic force microscopy (AFM) height image (a) of the surface topography of porous polytetrafluoroethylene (PTFE) shows the surface irregularities of the porous PTFE sheet, with average pore size of 1.86 µm and average particle size of 0.44 µm (b). Scale bar in (a) is 1 µm. AFM profiles of a typical particle (c) and pore (d).


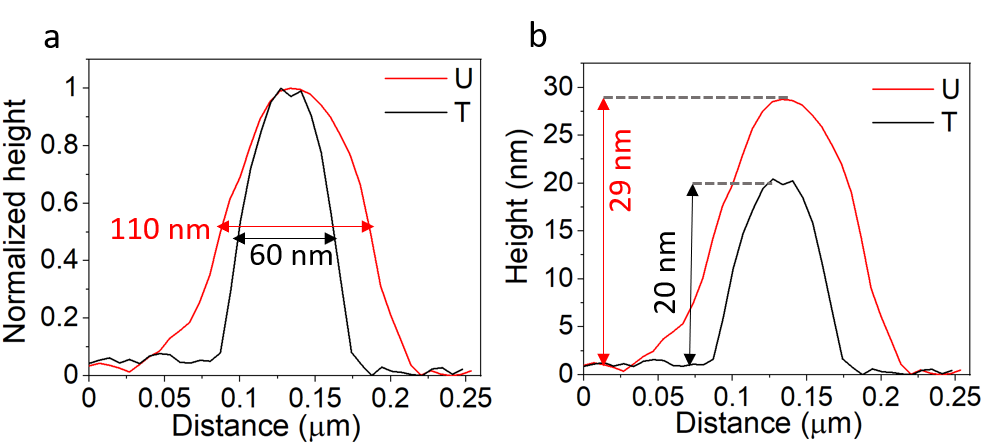


Figure S3. AFM height profiles of the untreated (U) and treated with 30 min direct pulsed UV laser irradiation (T) HCoV-229E virions. (a) Normalized height profiles show the comparison of the virion width. (b) Height profiles show the comparison of the virion height.


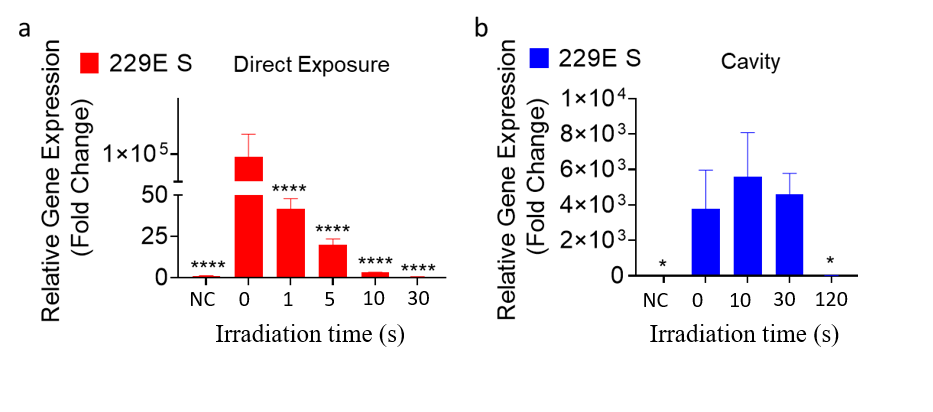


Fig S4. HCoV-229E S protein expression in Calu-3 cells. HCoV-229E virus was exposed to direct UVC laser (a) and cavity (b) for the indicated times. Calu-3 cells were treated 24 hours after seeding with the indicated groups of HCoV-229E (3 MOI). At 72 hours post-infection RNA was extracted and qPCR performed. Average fold change ±SEM, compared to the negative control (NC), is shown (N=3). A 1-Way ANOVA and Dunnett’s post hoc test was used to determine significance compared to 0 sec. * = p<0.05, ** = p<0.01, *** = P<0.001, ****= P<0.0001.


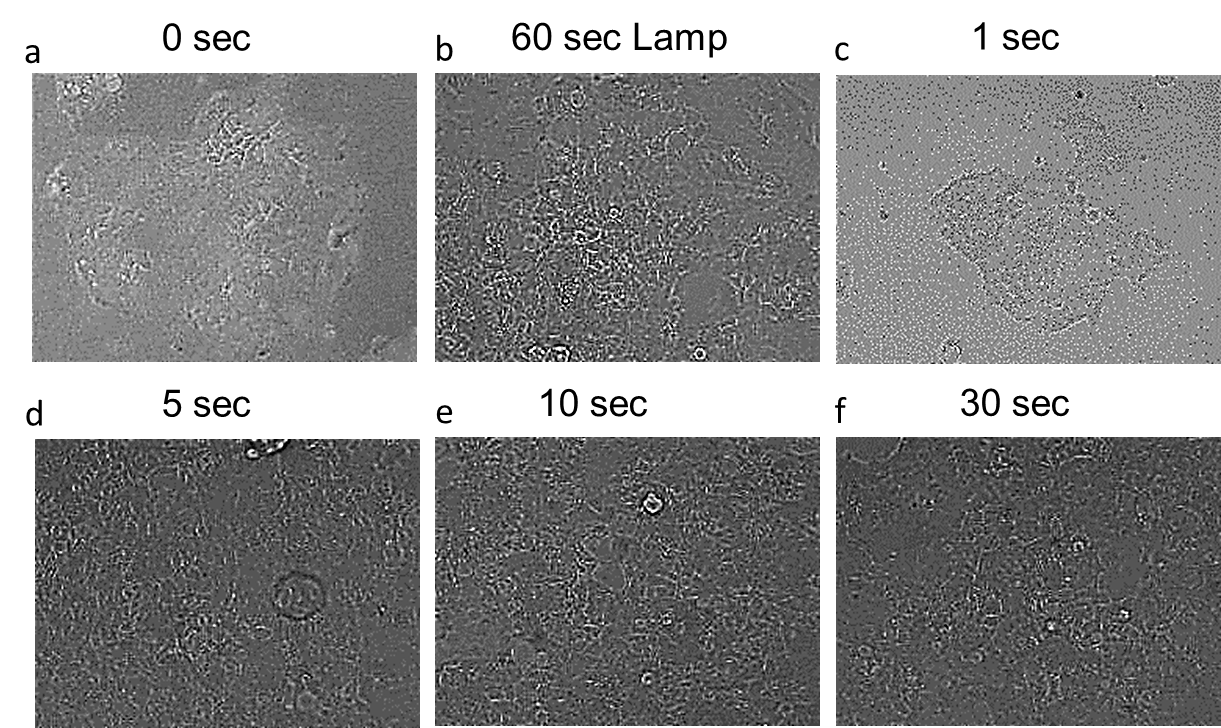


Fig S5. HCoV-229E virus was exposed to direct UVC laser light for the indicated times (a-f). Calu-3 cells were treated 24 hours after seeding with the indicated groups of HCoV-229E (3 MOI). Images were taken 72 hours post-infection using the Keyence BZ-X800 microscope. 200X.


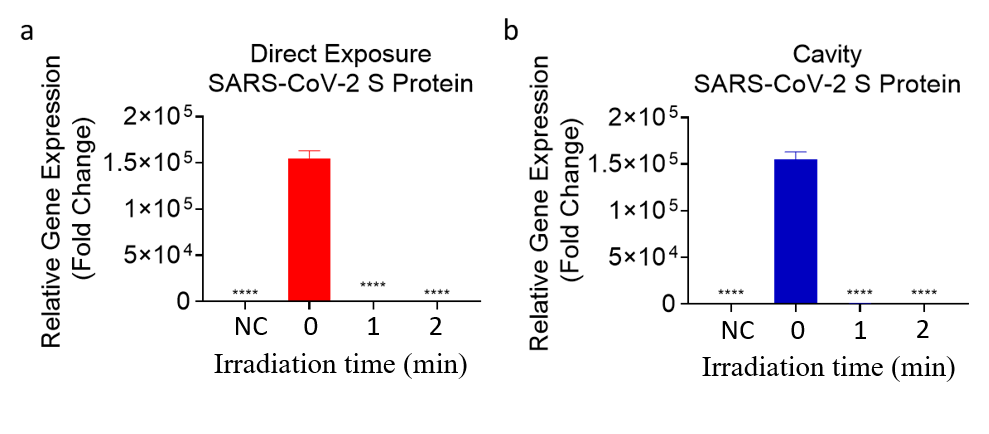


Fig S6. Sars-CoV-2 S protein expression in Calu-3 cells. SARS-CoV-2 virus was exposed to direct UVC laser (a) and cavity (b) for the indicated times. Calu-3 cells were treated 24 hours after seeding with the indicated groups of SARS-CoV-2 (0.1 MOI). At 72 hours post-infection RNA was extracted and qPCR performed. Average fold change ±SEM, compared to the negative control (NC), is shown (N=3). A 1-Way ANOVA and Dunnett’s post hoc test was used to determine significance compared to 0 sec. * = p<0.05, ** = p<0.01, *** = P<0.001, ****= P<0.0001.


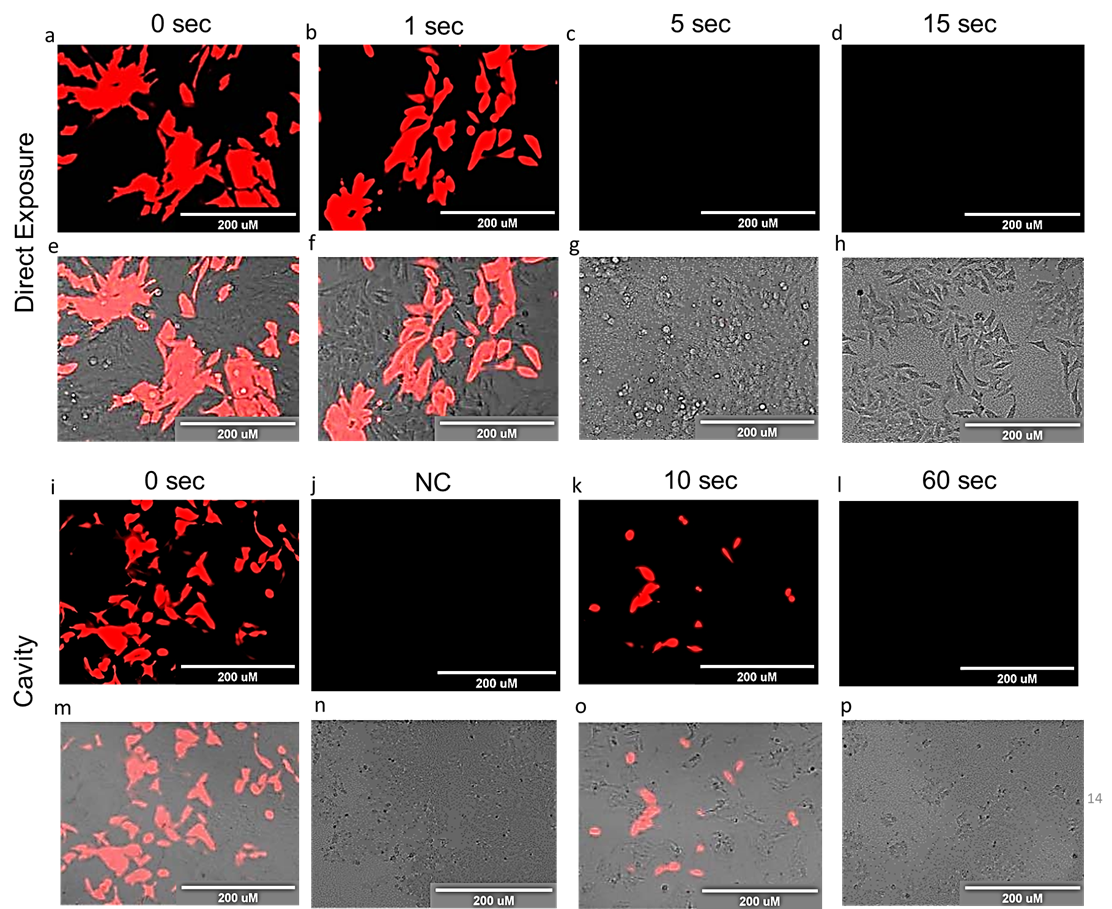


Fig S7. Direct UVC laser (a-h) and cavity (i-p) exposure of RSV-RFP virus. Hep-2 cells were treated 24 hours after seeding with the indicated groups of RSV-RFP (1 MOI) or the negative control (NC). Images were taken 72 hours post-infection using the Keyence BZ-X800 microscope. 200X.


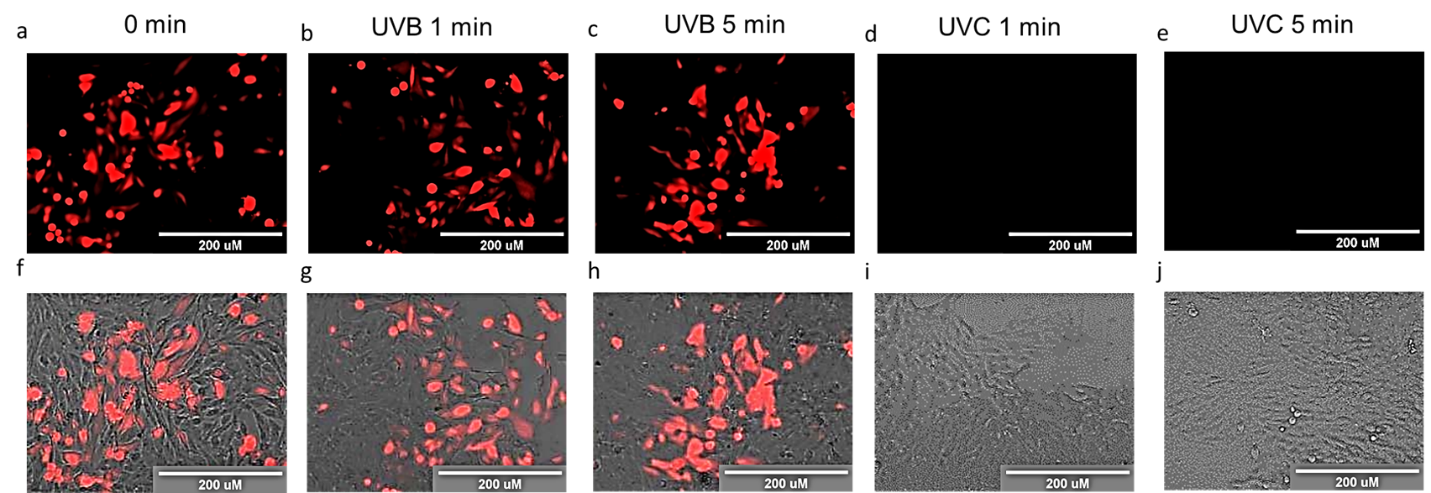


Fig S8. RSV-RFP virus was exposed to direct UVB or UVC laser light for the indicated times (a-j). Hep-2 cells were treated 24 hours after seeding with the indicated groups of RSV-RFP (1 MOI). Images were taken 96 hours post-infection using the Keyence BZ-X800 microscope. 200X.


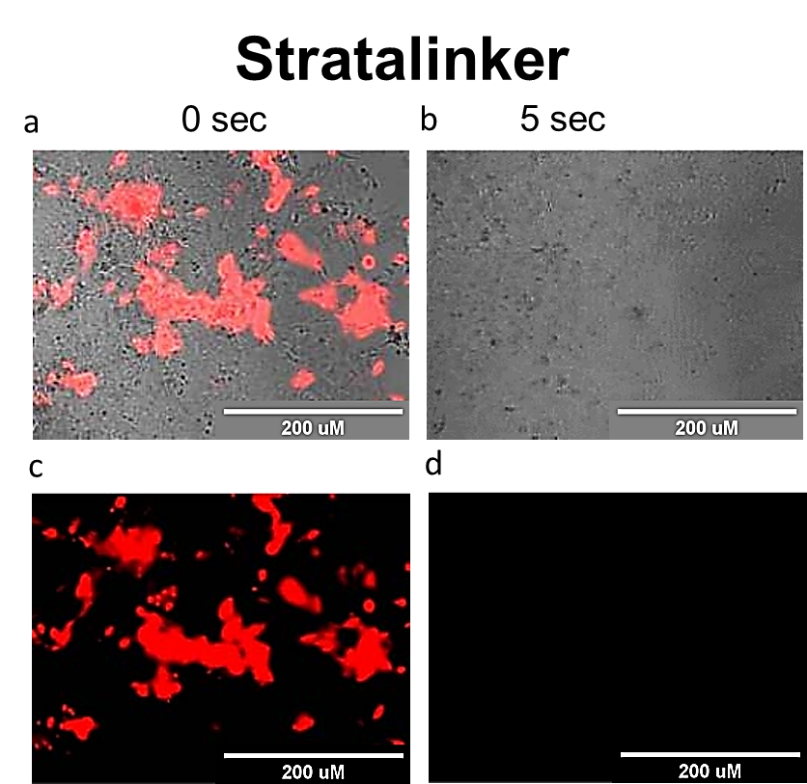


Fig S9. RSV-RFP virus was exposed to UVC lamp (Stratalinker) irradiation for the indicated times (a-d). Hep-2 cells were treated 24 hours after seeding with the indicated groups of RSV-RFP (1 MOI). Images were taken at 96 hours post-infection using the Keyence BZ-X800 microscope. 200X.


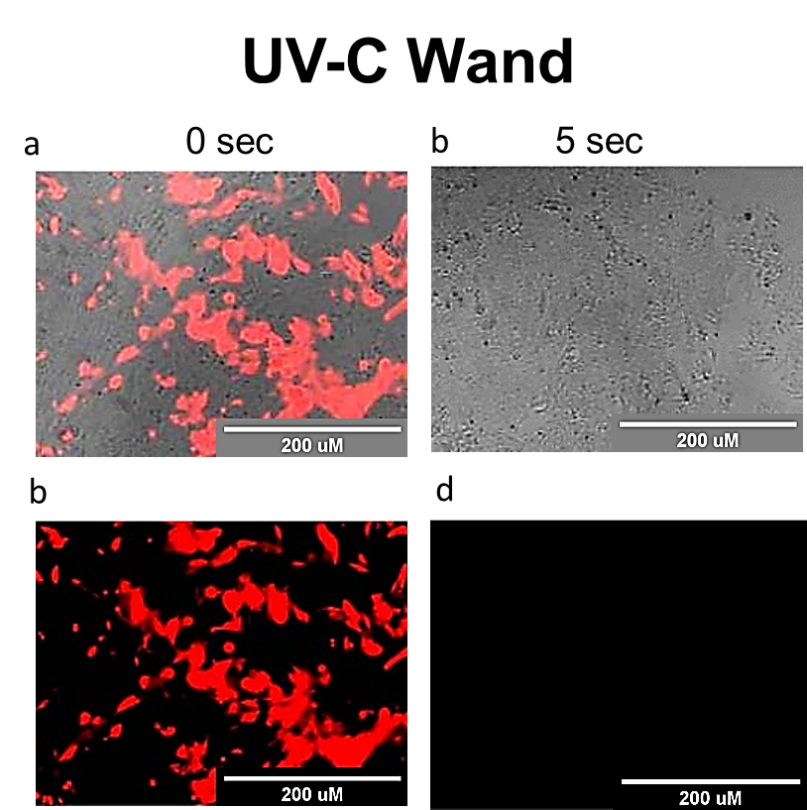


Fig S10. RSV-RFP virus was exposed to UVC lamp (Handheld Wand) for the indicated times (a-d). Hep-2 cells were treated 24 hours after seeding with the indicated groups of RSV-RFP (1 MOI). Images were taken at 96 hours post-infection using the Keyence BZ-X800 microscope. 200X.
